# Nrf2 and HSF-1 Pathway Activation via Hydroquinone-Based Proelectrophilic Small Molecules Is Regulated by Electrochemical Oxidation Potential

**DOI:** 10.1177/1759091415593294

**Published:** 2015-07-31

**Authors:** Takumi Satoh, Romain Stalder, Scott R. McKercher, Robert E. Williamson, Gregory P. Roth, Stuart A. Lipton

**Affiliations:** 1 Sanford-Burnham Neuroscience and Aging Research Center, La Jolla, CA, USA; 2 Department of Anti-Aging Food Research, School of Bioscience and Biotechnology, Tokyo University of Technology, Hachiouji, Japan; 3 Sanford-Burnham Medical Research Institute at Lake Nona, Orlando, FL, USA

**Keywords:** Nrf2, HSF-1, heat-shock proteins, phase 2 antioxidant enzymes

## Abstract

Activation of the Kelch-like ECH-associated protein 1/nuclear factor (erythroid-derived 2)-like 2 and heat-shock protein 90/heat-shock factor-1 signal-transduction pathways plays a central role in combatting cellular oxidative damage and related endoplasmic reticulum stress. Electrophilic compounds have been shown to be activators of these transcription-mediated responses through *S*-alkylation of specific regulatory proteins. Previously, we reported that a prototype compound (D1, a small molecule representing a proelectrophilic, *para*-hydroquinone species) exhibited neuroprotective action by activating both of these pathways. We hypothesized that the *para*-hydroquinone moiety was critical for this activation because it enhanced transcription of these neuroprotective pathways to a greater degree than that of the corresponding *ortho*-hydroquinone isomer. This notion was based on the differential oxidation potentials of the isomers for the transformation of the hydroquinone to the active, electrophilic quinone species. Here, to further test this hypothesis, we synthesized a pair of *para*- and *ortho*-hydroquinone-based proelectrophilic compounds and measured their redox potentials using analytical cyclic voltammetry. The redox potential was then compared with functional biological activity, and the *para*-hydroquinones demonstrated a superior neuroprotective profile.

## Introduction

**Figure 1.**
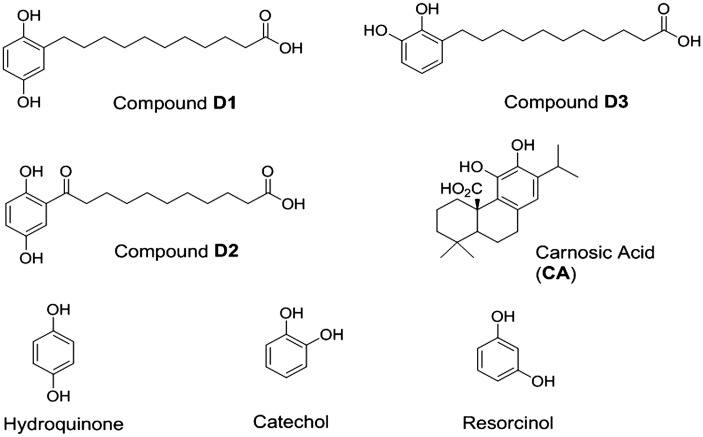
Chemical structures of the proelectrophiles evaluated in this study. The present study highlights compounds D1 (a) and D3 (b). CA (d) was evaluated as a neuroprotective compound in prior studies and is used here as a reference compound (Satoh et al., 2008a, 2008b). The compound notated as D2 (c) served as an inactive negative control for D1 in previous studies (Satoh et al., 2011). Note that D1 and D2 are *para*-hydroquinone isomers, while D3 and the natural product CA are *ortho*-hydroquinone isomers. CA = carnosic acid.

**Figure 2.**
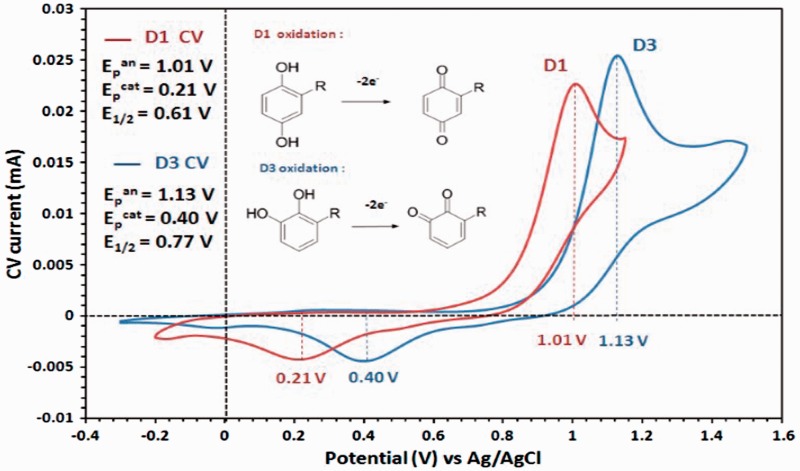
CV of D1 (red line) and D3 (blue line) recorded in 0.1 M (Bu)_4_NClO_4_ in acetonitrile (100 mV/s scan rates), with corresponding anodic peak (E_p_^an^), cathodic peak (E_p_^cat^), and half-wave (E_1/2_) potentials. Upon oxidation, the compounds adopt the oxidized quinone forms in a two-electron process. Both *para*- and *ortho*-hydroquinones are converted to electrophilic quinones, but the *meta*-hydroquinone isomer is not converted. *Para*- and *ortho*-hydroquinones, but not *meta*-hydroquinone, can activate the Nrf2 antioxidant pathway (Satoh and Lipton, 2007; Satoh et al., 2013). CA = carnosic acid; Nrf2 = nuclear factor (erythroid-derived 2)-like 2.

Living tissues and their associated cells maintain a delicate balance between reductive and oxidative processes to survive. Perturbation of this homeostatic redox balance is thought to significantly contribute to various disorders, including Alzheimer’s and Parkinson’s diseases (Hara and Snyder, 2007; Satoh and Lipton, 2007; Bredesen, 2008; Kim et al., 2008; Nakamura and Lipton, 2009). Recently, considerable attention has focused on electrophilic and proelectrophilic drugs (PEDs) as well as their related analogues because of their ability to activate cellular defense systems (Satoh et al., 2006; Calabrese et al., 2010; Groeger and Freeman, 2010; Satoh et al., 2013). PEDs such as carnosic acid (CA; Figure 1) are natural products found in herbs such as rosemary and sage (Aruoma et al., 1992; Satoh et al., 2008a, 2008b). In addition, related natural product-inspired hydroquinone-based synthetic compounds (i.e., D1 and D3; Figure 2) have recently been reported by our team and were shown to be bioactive (Satoh et al., 2011). Importantly, CA and D1 (Figure 1), examples of PEDs, themselves are not electrophilic until they are activated at a site of tissue injury undergoing oxidative stress (Satoh et al., 2011).

PED activation under conditions of oxidative stress occurs because quinone formation is influenced by the cellular redox state, and in particular the Cu^2+^/Cu^+^ recycling system (Wang et al., 2010; Satoh et al., 2013). The redox-active transition metal copper can catalyze oxidative activation of a number of phenolic compounds via Cu^2+^/Cu^+^ cycling (Bensasson et al., 2008; Satoh et al., 2009; Wang et al., 2010; Johansson, 2012). Under normal conditions, PED hydroquinones are very slowly oxidized to the quinone form, whereas this rate is greatly increased in the presence of Cu^2+^/Cu^+^ recycling (Wang et al., 2010; Satoh et al., 2013). In addition, after conversion to the active form, PEDs display superiority to classical antioxidant molecules because of their sustained action and amplification via the nuclear factor (erythroid-derived 2)-like 2 (Nrf2) and heat-shock factor-1 (HSF-1) transcription-mediated signaling pathways (Satoh et al., 2013). These dynamic processes afford the potential for generating a pathologically activated therapeutic (or PAT) drug (Lipton, 2004, 2006, 2007). Importantly, we have also shown that several PEDs display an excellent absorption, distribution, metabolism, excretion, toxicity, and pharmacokinetic profile in the central nervous system, making them strong drug candidates (Satoh et al., 2008a; Rezaie et al., 2012; Satoh et al., 2013).

First, we focused on the Kelch-like ECH-associated protein 1 (Keap1)/Nrf2 pathway as one of the targets of PEDs for neuroprotection (Satoh et al., 2008b, 2011, 2013). After conversion to electrophilic quinones, PEDs activate the Keap1/Nrf2 pathway (Satoh and Lipton, 2007; Satoh et al., 2008b, 2013). We have shown that it is possible to take advantage of this intracellular mechanism of electrophilic activation to develop a novel strategy for drug development against neurodegenerative diseases. As an illustration, CA activates the Nrf2 transcriptional pathway and protects various cells and organs against redox stress (Takahashi et al., 2009; Kosaka et al., 2010; Mimura et al., 2010; Tamaki et al., 2010; Maruoka et al., 2011; Satoh et al., 2011; Rezaie et al., 2012; Yanagitai et al., 2012). We have developed a potential strategy for clinical translation of this work based on the following chemical principles (Satoh and Lipton, 2007; Satoh et al., 2013).

1. Novel PEDs and related analogues are better tolerated than electrophiles, in part because electrophilic compounds can deplete glutathione in healthy, unstressed cells (Bensasson et al., 2008; Satoh et al., 2009; Wang et al., 2010; Johansson, 2012).
2. PEDs combat the oxidative stress that converts them to electrophiles through activation of the Nrf2 pathway (Bensasson et al., 2008; Satoh et al., 2009; Wang et al., 2010; Johansson, 2012).

Second, we focused on the heat-shock protein (HSP)90/HSF-1 pathway as one of the targets of PEDs for neuroprotection (Satoh et al., 2011, 2013). HSP90/HSF-1 (Zou et al., 1998; Morimoto, 2008; Takii et al., 2010), like Keap1/Nrf2 (Talalay, 2000; Itoh et al., 2004; Zhang et al., 2011), provides cell protection through activation of endogenous gene networks involved in antioxidant response element (ARE) defense (Satoh and Lipton, 2007; Satoh et al., 2013). Several activators of Nrf2 via reaction with Keap1 also covalently bind to cysteine residues of HSP90 to activate HSF-1 and other HSPs (Satoh et al., 2011; Zhang et al., 2011) through binding to AREs and heat-shock factor response elements (HSE), respectively, to provide neuroprotection against oxidative and nitrosative insults (Satoh et al., 2011; Zhang et al., 2011). Thus, *S*-alkylation of critical cysteine residues by electrophilic compounds can activate both of these transcriptional pathways, representing what has been called an electrophilic counterattack response (Satoh et al., 2006; Satoh and Lipton, 2007; Groeger and Freeman, 2010; Satoh et al., 2013). Because the capacity of neurons to protect themselves from redox insults can be easily overwhelmed, such backup systems are important homeostatic regulation processes that insure the continuation of normal neuronal signaling (Satoh et al., 2006; Satoh and Lipton, 2007; Groeger and Freeman, 2010; Satoh et al., 2013). D1 is a novel proelectrophilic compound that activates both the Nrf2 and HSF-1 pathways and can thus protect against both oxidative and endoplasmic reticulum (ER) stress (Satoh et al., 2011; Zhang et al., 2011).

Importantly, potential clinical relevance for the use of PEDs *in vivo* has been obtained in models of age-related macular degeneration (AMD; Rezaie et al., 2012; Satoh et al., 2013) and cerebrovascular disease (stroke; Satoh et al., 2008b). While antioxidants were generally believed to have some effect in preventing AMD, their actions are fairly marginal (Prasad, 2009; Cano et al., 2010; Ramkumar et al., 2010). The use of antioxidants is not very effective because tissue penetrance is not reliable and often the action is not sustained. Similarly, while tissue plasminogen activator can dissolve clots, no effective neuroprotective treatment has been proven for stroke damage in the brain (Satoh et al., 2008a, 2008b). Most importantly, the novel PEDs, such as D1 described in this article, increase the chances of producing clinically tolerated therapeutics because they are only changed to the active state by oxidative insult at the site of impending injury (Satoh et al., 2013).

Based on these inherent attributes and the potential clinical relevance of PEDs, we investigated their effects on various types of cell lines. For example, because of the potential involvement of oxidative stress in AMD, we used the human apical retinal pigment epithelial cell line, ARPE-19 (Wada et al., 2001; Kim et al., 2003; Mandel et al., 2009). Because of the potential involvement of oxidative stress in Alzheimer’s disease, vascular dementia, and stroke, we used the mouse hippocampal cell line, HT-22 (Hara and Snyder, 2007; Satoh and Lipton, 2007; Bredesen, 2008; Kim et al., 2008; Nakamura and Lipton, 2009). Glutamate may be directly toxic to cultured neuronal cells via two different processes (Tan et al., 1998; Sagara et al., 2002). The first pathway is mediated by glutamate receptors. The second pathway is activated by a reduction in intracellular glutathione levels, leading to an imbalance in the homeostasis of the cell’s redox state (Tan et al., 1998; Sagara et al., 2002). This second pathway can be blocked by the addition of antioxidants. In particular, glutamate toxicity of HT22 cells has been used to model oxidative stress-induced cell death in hippocampal neurons (Tan et al., 1998; Sagara et al., 2002).

The objective of this study was to determine which isomer (*para-* vs. *ortho-*hydroquinone) could provide maximal activation of the Nrf2/ARE and HSF-1/HSE pathways. We sought to combine chemical and biological perspectives on this issue to define the best chemical structure of an electrophilic core for use as a PED. To test the *para*-hydroquinone electrophilic core of D1 versus an isomeric *ortho*-analogue, we synthesized compound D3 (the *ortho*-hydroquinone variant of D1) having the same chemical scaffold (Figure 1). We then compared the *para*- and *ortho*-forms in terms of chemical and biological actions by monitoring oxidation potential, transcriptional activation, induction of phase 2 enzymes and HSPs, as well as protection against oxidative and ER stress.

## Materials and Methods

### Chemicals and Antibodies

Antibodies and reagents were obtained as follows: anti-heme oxygenase-1 (HO-1) polyclonal rabbit IgG (OSA-150, Assay Design, Ann Arbor, MI), anti-NADPH quinone oxidoreductase1 (NQO1) polyclonal rabbit IgG (2618-1, Epitomics, Cambridge, MA), anti-HSP70 monoclonal mouse IgG (200-301-A27, Rockland, Pottstown, PA), IRDye 800CW goat anti-rabbit (green fluorescent; LI-COR, Lincolon, NE, catalogue number 926-32211), and IRDye 680LT goat anti-mouse (red fluorescent; LI-COR, Lincolon, NE, catalogue number 926–68020). Other reagents including dimethylsulfoxide (DMSO), sodium glutamate, fluorescein diacetate (FDA), hydrogen peroxide (HP), tunicamycin (TM), and Hoechst 33 258 stain were obtained from Sigma (St Louis, MO). The chemical synthesis of D1 has been described previously (Satoh et al., 2011). Synthesis of analogues D1 and D3 are described later.

### General Procedure for the Synthesis of Compounds, D1 and D3

To a 20-ml microwave vial was added methyl 10-bromodecanoate (1.0 equiv.) and triphenylphosphine (2.0 equiv.). The mixture was placed under an argon atmosphere and subjected to heating via microwave irradiation at 100℃ for 45 min. The resulting mixture was then cooled to room temperature, followed by addition of anhydrous tetrahydrofuran (THF; 10 ml) and mild heating and vortexing until a solution was formed. To the resulting phosphonium salt solution was then added 1.0 M sodium bis-trimethylsilylamide (1.0 equiv.) dropwise over a 1-min period. This mixture was stirred at 0℃ in an ice/brine bath for 45 min followed by addition of the corresponding *ortho*- or *para*-dimethoxybenzaldehydes (1.48 M in THF). The reaction was allowed to proceed, with stirring, at room temperature over 16 hr. The reaction product was then concentrated and reconstituted in 20 ml of ethyl acetate (EtOAc)/water (50/50 v/v), after which the layers were separated. The aqueous layer was extracted with EtOAc (3 × 8 ml) and combined with the organic layer, and the mixture was then dried over MgSO_4_. The product was subsequently filtered, concentrated, and subjected to SiO_2_ flash column chromatography (Biotage SP4 system, Uppsala, Sweden) using 5% to 18% EtOAc to afford the olefinic esters as a colorless oils (46%–68%). These intermediates were used directly in the next step.

To a 5-ml round-bottom flask containing the mixed olefinic ester intermediates was added anhydrous methanol (0.1 M) followed by addition of 10% Pd/C (0.1 equiv.). The reaction was placed under a hydrogen atmosphere and stirred vigorously for 16 hr. After such time, the mixture was filtered through a plug of celite/SiO_2_ and concentrated to yield the crude aliphatic ester in quantitative yield. To the crude ester were added equal amounts of THF and 2 M LiOH_(aq)_ solution (4 equiv.), and this mixture was then stirred at 40℃ overnight. The reaction product was concentrated, dissolved in EtOAc, and then acidified (to pH 1–2) via addition of 1 N HCl. The layers were separated, and the acidic aqueous layer was extracted three times with EtOAc. The combined extracts were dried over Na_2_SO_4_, filtered through a SiO_2_ solid phase extraction cartridge, and concentrated to yield the crude acids affording colorless oils. The oils were diluted through addition of anhydrous CH_2_Cl_2_ and cooled to −78℃ in a dry ice/acetone bath. To this solution was added 1 M BBr_3_ (2.1 equiv.) in CH_2_Cl_2_, and this mixture was allowed to gradually return to room temperature over a period of 4 hr. Thereafter, the temperature was reduced to 0℃. The reaction was quenched with deionized water and extracted three times with EtOAc. The combined extracts were dried over Na_2_SO_4_, filtered over celite, and concentrated. The crude product was dissolved in methanol and subjected to purification via preparative liquid chromatography/mass spectrometry using mass-directed fractionation. Combined fractions were concentrated using a Biotage V-10 evaporation system to afford compounds D1 or D3 as white/off-white solids (20%–35% isolated yield over three steps).

#### Compound D1

^1^H-nuclear magnetic resonance (NMR; 500 MHz, deuterated methanol, CD_3_OD) δ ppm: 6.57 (d, *J* = 8.5 Hz, 1H), 6.52 (d, *J* = 2.9 Hz, 1H), 6.43 (dd, *J* = 8.5, 3.0 Hz, 1H), 2.54 − 2.46 (m, 2H), 2.26 (t, *J* = 7.4 Hz, 2H), 1.64 − 1.51 (m, 4H), 1.39 − 1.25 (m, 12H). ^13^C-NMR (125 MHz, deuterated methanol, CD_3_OD) δ ppm: 178.3, 151.1, 149.2, 131.5, 117.7, 116.7, 114.0, 35.4, 31.3, 31.1, 30.8, 30.7, 30.7, 30.7, 30.5, 30.4, 26.4. High-resolution mass spectrometry calculated *m/z* for C_17_H_26_O_4_ [M-H]^−^= 293.1758; *m/z* found 293.1756.

#### Compound D3

^1^H NMR (500 MHz, deuterated methanol, CD_3_OD) δ ppm: 6.62−6.52 (m, 3H), 2.61−2.52 (m, 2H), 2.26 (t, *J* = 7.5 Hz, 2H), 1.64−1.53 (m, 4H), 1.37−1.25 (m, 12H). ^13^C NMR (125 MHz, deuterated methanol, CD_3_OD) δ ppm: 178.5, 146.0, 144.4, 130.9, 122.1, 120.2, 113.8, 35.6, 31.2, 31.1, 30.8, 30.7, 30.7, 30.7, 30.5, 30.4, 26.4. High-resolution mass spectrometry calculated *m/z* for C_17_H_26_O_4_ [M-H]^− ^= 293.1758; *m/z* found 293.1757.

### Cyclic Voltammetry Experimental Protocol

All experiments were performed using an electrochemical analyzer (CH Instruments, Austin, TX, model 600 E), glassware (cells), hardware, and electrodes (glassy carbon working, platinum counter, Ag/AgCl reference). All glassware was cleaned with concentrated nitric acid and rinsed with deionized water prior to experimentation. Prior to and between cyclic voltammetry (CV) scans, the glassy carbon electrode was polished with 0.05-micron alumina, the platinum wire auxiliary electrode was cleaned in 0.1 M H_2_SO_4_, and the Ag/AgCl reference electrode was stored in 1.0 M KCl. All substrates were prepared as 1 mM solutions in 0.1 M tetrabutylammonium perchlorate in anhydrous acetonitrile and degassed under argon for 10 min prior to and between CV scans. Anodic and cathodic potentials were recorded after at least 10 cycles to insure reproducibility, except for resorcinol, which was recorded for the first cycle due to rapid current decrease upon subsequent cycles (Nasr et al., 2005; Astudillo et al., 2007; Nematollahi and Mohammadi-Behzad, 2009).

### Cell Culture (ARPE-19 Cells)

To study the biochemistry and molecular biology of oxidative stress in retina, several investigators have used *in vitro* culture of ARPE-19 cells. For example, exposure of ARPE-19 cells to HP is known to induce apoptosis and has thus been used as an *in vitro* model of retinal degeneration triggered by oxidative stress (Satoh et al., 2011). The cells were maintained in 10-cm dishes containing Dulbecco’s modified Eagle’s medium (DMEM) supplemented with 10% fetal calf serum. The cells were introduced into wells of a 24-well plate at a density of 1 × 10^5^ cells/cm^2^ and incubated for 24 hr. The medium was then changed to serum-free medium containing the designated concentrations of the test compounds, and the cultures were incubated for 24 hr. Then, HP or TM was added, and the cells were incubated for 4 hr or 24 hr, respectively. Finally, the cells were stained with FDA (1 µM) and Hoechst 33258 (5 µg/ml) and observed by epifluorescence microscopy.

### Reverse Transcription-Polymerase Chain Reaction

For reverse transcription-polymerase chain reaction (RT-PCR) analysis, total RNA was obtained by use of TRIZOL Reagent (Invitrogen, Waltham, MA) from ARPE-19 cells that had been incubated with vehicle (DMSO), D1 (5 µM), or D3 (5 µM) in serum-free medium for 24 hr (Satoh et al., 2000, 2003; Sasaki et al., 2011, 2013). Total RNA (1 µg) from each source was exposed to Superscript III (Invitrogen) in the presence of RNasin (20 U), random hexamers (2.5 µM), dNTPs, and the supplied reverse transcription buffer. The reaction (20 µl) was allowed to continue for 15 min at 42℃. A volume of 1/100th of this mixture from each source was then subjected to PCR conducted with the appropriate primer sets. At completion of the PCR, 10 µl of the PCR products were mixed with 2 µl of loading buffer, and this mixture was electrophoresed in 1.5% agarose gel in the presence of 0.5 µg/ml ethidium bromide. The amplified DNA fragments were visualized with UV detection.

RT-PCR analysis was performed as described previously (Satoh et al., 2000, 2003; Sasaki et al., 2011, 2013) with the following primers (number of PCR cycles and size of PCR product in parentheses):

5′-TGA CTG ACT ACC TCA TGA AG-3′ (F) and
5′-TTG CCA ATG GTG ATG ACC TG-3′ (R) for β-actin (22 cycles, 202 bp);
5′-GAG TTG CAG CTG CTG AG-3′ (F) and
5′-GCA TGC CTG CAT TCA CAT G-3′ (R) for ho-1 (24 cycles, 233 bp);
5′-CTC CAT GTA CTC TCT GCA AG -3′ (F) and
5′-GTG GTG TCT CAT GAG TGT GC -3′ (R) for nqo1 (30 cycles, 203 bp); and
5′-AGA TTC ATG ACG TCG TCC TG-3′ (F) and
5′-GGA TGC CAT TAG CAT CAA TG-3′ (R) for hsp70 (28 cycles, 468 bp).

### Western Blot Analysis

ARPE-19 cells were incubated for 24 hr with vehicle (DMSO), D1 (5 µM), or D3 (5 µM) in 10-cm dishes containing serum-free DMEM (Satoh et al., 2000, 2003; Sasaki et al., 2011, 2013). The cells were lysed in buffer (M-PER mammalian protein extract reagent, catalog #78503, Thermo Scientific, Waltham, MA) supplemented with a protease inhibition cocktail (Complete Protease Inhibitor Cocktail Tablets; catalog #11836170001, Roche, Waltham, MA). Total cell lysates (10 µg each) were separated by sodium dodecyl sulfate-polyacrylamide gel electrophoresis and then transferred onto polyvinylidene fluoride membranes. HO-1, NQO1, HSP70, and glyceraldehyde 3-phosphate dehydrogenase (GAPDH) were detected with specific antibodies, and the signals were detected using peroxidase-conjugated secondary antibodies. The protein signals were enhanced by use of a chemiluminescence assay (ECL Western blotting; Amersham Pharmacia, Piscataway, NJ).

### Luciferase Assay

We used pGL-GSTYa ARE core-luciferase (Satoh et al., 2008, 2011) and ptK-hHSP70-luc (Takii et al., 2010) for assessment of transcriptional activation via ARE and HSE, respectively. ARPE-19 cells were seeded at a density of 1 × 10^5^ cells/cm^2^ into wells of a 48-well plate containing 1 µg of plasmid DNA plus Lipofectamine 2000 (Invitrogen), and then incubated for 5 hr in PBS containing 1 µg of plasmid DNA plus Lipofectamine 2000 (Invitrogen). The cells were then washed in PBS and incubated for another 24 hr with vehicle, D1 (5 or 10 µM), or D3 (5 or 10 µM). Firefly luciferase activity in cell lysates was measured with a luminometer for reporter gene assays (Promega, Madison, WI). Transfection efficiency was normalized to β-galactosidase activity assessed by co-transfection with pSV-β-gal (Promega). For reporter gene assays, cells were transfected with 1 µg of the reporter construct (pGL-GSTYa ARE core-luciferase or ptK-hHSP70-luc) and 0.2 µg pSV-β-gal for 1 hr. Cells were then washed in PBS and incubated in serum-free culture medium for another 24 hr with vehicle, D1 (5 or 10 µM), or D3 (5 or 10 µM). Firefly luciferase activity and β-galactosidase activity in cell lysates were measured by using a luciferase system and β-galactosidase enzyme assay system, respectively (Promega).

### Culture of HT22 Cells

Mouse hippocampal HT22 cells were maintained in 10-cm dishes containing DMEM supplemented with 10% fetal calf serum (Satoh et al., 2000, 2003; Sasaki et al., 2011, 2013). The cells were seeded onto 24-well plates at a density of 8 × 10^4^ cells per well in 500 μl of serum-containing DMEM. One hour after seeding, the cultures were incubated for 1 hr with vehicle (DMSO) or various concentrations of D1 or D3. One hour later, the cells were exposed to 5 mM glutamate for 24 hr to induce oxidative damage. Subsequently, viability of the HT22 cells was determined using 3-(4,5-dimethylthiazol-2-yl)-2,5-diphenyl tetrazolium bromide (MTT) reduction, as described elsewhere (Satoh et al., 2000, 2003; Sasaki et al., 2011, 2013). Cell death was also measured by the lactate dehydrogenase (LDH) cytotoxicity detection kit (TAKARA, Otsu, Shiga, Japan), which quantitatively measures the release of LDH into the medium following cell lysis or cell death. This assay was used as described by the manufacturer.

### Statistical Analysis

Results are presented as the mean ± *SD*. Analysis of variance with an appropriate post hoc test was performed for multiple comparisons and a Student’s *t* test for comparison of two samples. A *p* value ≤ .05 was considered significant.

## Results

### Redox State of Compounds

The redox behavior of D1 and D3 was analyzed using CV in solution. Their voltammograms were compared with that of 1,2-dihydroxybenzene (catechol), 1,3-dihydroxybenzene (resorcinol) and 1,4-dihydroxybenzene (hydroquinone), which were measured as control compounds in parallel to that of D1 and D3 (Nasr et al., 2005; Astudillo et al., 2007; Nematollahi and Mohammadi-Behzad, 2009). The CVs of catechol and hydroquinone were recorded in the−0.5 V to 1.5 V potential range versus Ag/AgCl, where one anodic peak (oxidation to quinone form) and one cathodic peak (reduction back to the hydrogenated form) are observed. Table 1 summarizes the potential values for all compounds. Redox processes in 0.1 M tetrabutylammonium perchlorate in acetonitrile electrolyte are not reversible because the anodic and cathodic peaks are separated by over 600 mV. This was expected and consistent with values reported in the literature for these substrates/electrolyte combinations, and in particular hydroquinone is confirmed to oxidize more readily than catechol by 0.1 V. The CVs of resorcinol show only one oxidation peak at 0.3 V to 0.4 V higher potential than the other two isomers, which is consistent with the lack of electron delocalization on the nonconjugated *meta*-substitution pattern of the molecule (Nasr et al., 2005; Astudillo et al., 2007; Nematollahi and Mohammadi-Behzad, 2009). The CVs of the oxidation processes of D1 and D3 are shown in Figure 2. The anodic peak for D1 is found at 1.01 V, and that of D3 is recorded at 1.13 V. With cathodic peaks at 0.21 V and 0.40 V, respectively, the half-wave potentials for D1 and D3 are at 0.61 V and 0.77 V. This is consistent with the values found for the hydroquinone and catechol model compounds (Table 1 and Figure 1) and confirms that all redox processes involving the oxidation of D1 occur at 0.1 V to 0.2 V lower potentials than D3, that is, D1 is oxidized more readily than D3.

**Table 1.** Anodic (Oxidation) and Cathodic (Reduction) Peak Potentials and Corresponding Half-Wave Potentials From the CVs of D1, D3, and the Unsubstituted Control Compounds Shown in Figure 1.

Compound	E_p_^anode^ (V)	E_p_^cathode^ (V)	E_1/2_ (V)
Hydroquinone	1.06	0.33	0.70
Resorcinol	1.49	n/a	n/a
Catechol	1.16	0.55	0.86
D1	1.01	0.21	0.61
D3	1.13	0.40	0.77

*Note*. CVs recorded using 0.1 M (Bu)_4_NClO_4_ in acetonitrile with 100 mV/s scan rates versus an Ag/Ag^+^ reference electrode. CV = cyclic voltammetry.

### Activation of the Nrf2 and HSF-1 Pathways

**Figure 3.**
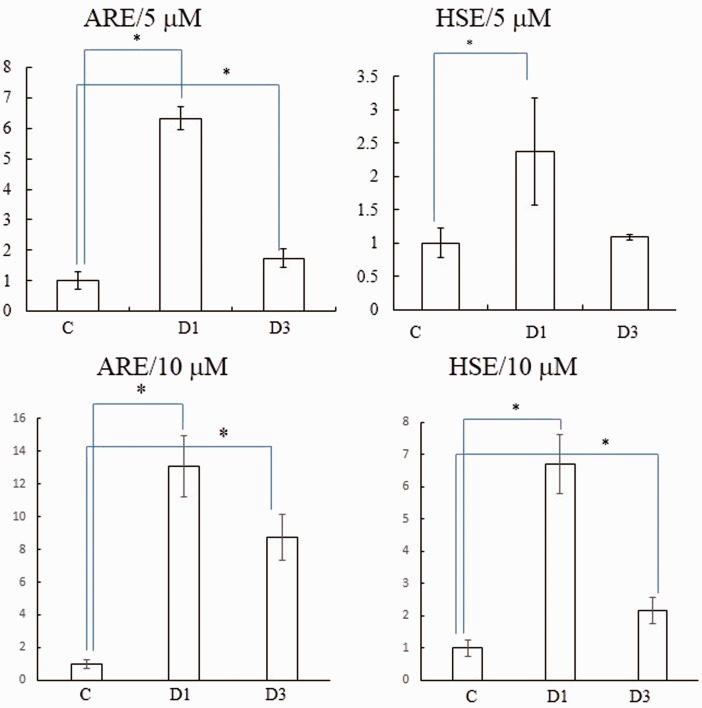
Transcriptional activation of ARE and HSE. Retinal pigment epithelial ARPE-19 cells were plated at 1 × 10^5^ cells/cm^2^, incubated for 24 hr, and then transfected with DNAs (ARE- or HSE-luciferase construct). After a 5-hr incubation in serum-containing medium, the medium was changed to serum-free medium containing vehicle (DMSO) versus D1 or D3 (5 µM or 10 µM). Cell lysates were obtained after 24 hr incubation and subjected to the luciferase assay. Values are the mean ± *SD*; *p < .05. ARE = antioxidant response element; HSE = heat-shock factor response element; ARPE = human apical retinal pigment epithelial cell line; DMSO = dimethylsulfoxide.

We hypothesized that D1 activated Nrf2 and HSF-1 more than D3 based on the redox potential and ease of oxidation to the cysteine-reactive quinone form. To confirm this notion, we performed luciferase assays in ARPE-19 cells transfected with plasmid DNAs under the transcriptional control of the ARE or HSE (Figure 3). D1 (5 µM) significantly activated both transcriptional elements, indicating that D1 can stimulate both the ARE and HSE systems. In contrast, the *ortho*-isomer D3 (5 µM) activated the Nrf2/ARE pathway to a lesser extent than that of the *para*-isomer. At 10 µM, D1 was still more potent than D3. D3 at 10 µM but not 5 µM activated the HSE transcriptional element. These observations are consistent with the notion that the *para-*isomer (D1) activates the Keap1/Nrf2 and HSP90/HSF-1 pathways more effectively than the *ortho-*isomer (D3).

### Induction of Phase 2 Enzymes

**Figure 4.**
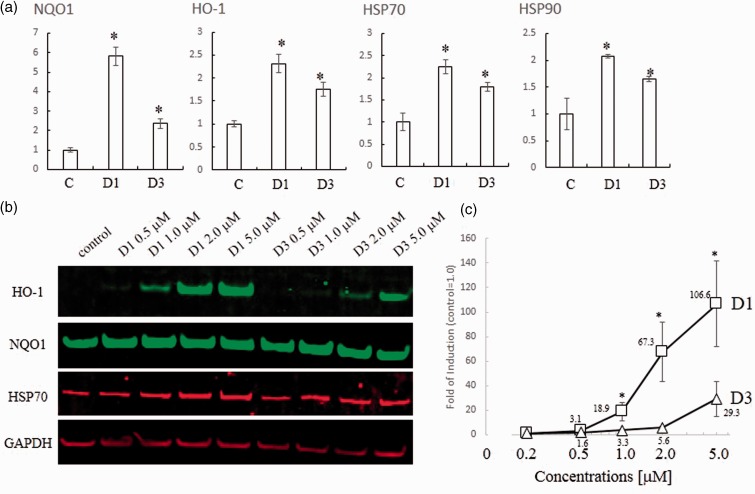
Induction of phase 2 enzymes and HSPs. (a) PCR analysis of phase 2 and HSP genes induced by D1 or D3. Total RNA was extracted from ARPE cells treated with 5 µM D1 or D3 for 24 hr in serum-free medium. RT-PCR was performed using cDNA template with the specific primers listed in the Materials and Methods section mRNA levels were quantified by densitometry of qPCR band intensity after 28 cycles (*n* = 3). All genes were normalized to β-actin expression. *Significantly different (*p* < .05) from control. (b) Western blot analysis of dose-dependent induction of HO-1, NQO1, HSP70, and GAPDH proteins by the indicated concentrations of D1 or D3. Cell lysates were prepared, and 10 µg protein/lane of protein was subjected to sodium dodecyl sulfate-polyacrylamide gel electrophoresis, after which the proteins were detected by use of specific antibodies. (c) HO-1 was normalized to GAPDH by taking the ratio of their densitometric values on immunoblots. *Significantly different (*p* < .05) between D1 and D3. HSP = heat-shock protein; RT-PCR = reverse transcription-polymerase chain reaction; ARPE = human apical retinal pigment epithelial cell line; HO-1 = heme oxygenase-1; NQO1 = NADPH quinone oxidoreductase1; GAPDH = glyceraldehyde 3-phosphate dehydrogenase.

We hypothesized that the biological activity of compounds D1 and D3 would be closely related to their ability to activate the Nrf2/ARE pathway. The previous DNA microarray study showed that compound D1 induced expression of HSPs in addition to phase 2 enzymes. Incubation with D1 resulted in activation of Nrf2 and HSF-1 transcriptional elements, thus inducing phase 2 enzymes and HSPs, respectively (Satoh et al., 2011). In this manner, D1 protected neuronal cells from both oxidative and ER-related stress. First, in this article, to compare the induction of the genes encoding phase 2 enzymes and HSPs by 5 µM D1 and D3, we performed an RT-PCR analysis using primers for the *ho-1* and *nqo1* phase 2 genes, and for *hsp70* and *hsp90* (Figure 4(a)). D1 significantly induced each of these genes, although the magnitude of induction varied from gene to gene. Induction by D3 was significantly weaker than that by D1. Next, we confirmed the induction of HO-1, NQO1, and HSP70 at the protein level by performing immunoblot analysis (Figure 4(b)). D1 induced HO-1 in a dose-dependent manner, whereas D3 did so only weakly, as confirmed by quantitative analysis (Figure 4(c)). While D1 and D3 induced the expression of NQO1 and HSP70 proteins, the basal levels of expression were already high in this cell line. Taken together, these data suggest that D1 induced both phase 2 enzymes and HSPs both more potently and efficaciously than D3.

### Protective Effects in ARPE-19 Cells

**Figure 5.**
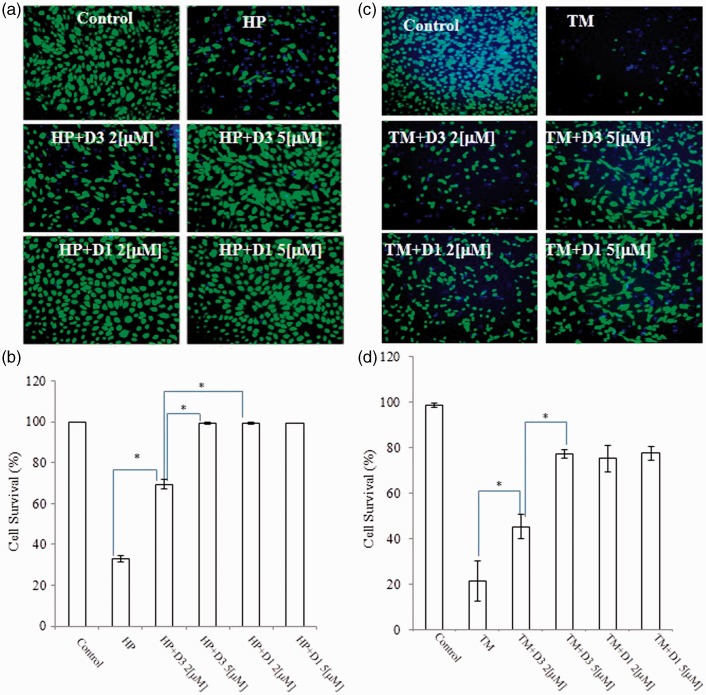
Protective effects of various PEDs on ARPE-19 cells. (a and c) ARPE-19 cells were plated at 1 × 10^5^ cells/cm^2^ and incubated for 24 hr. Then, the medium was changed to serum-free medium containing vehicle (DMSO), D1 or D3, and the cells were incubated for an additional 24 hr. Thereafter, they were incubated with 1 mM HP, 3 µM TM, or vehicle for 4 hr, and then stained with fluorescein diacetate (FDA, green) and Hoechst dye 33,258 (blue; Satoh et al., 2011). Because FDA (green) and Hoechst 33,258 (blue) originate from living cells and all cells, respectively, *blue-only* cells are dead cells (often morphologically with a shrunken, bright nucleus signifying apoptosis) and blue + green cells are living cells. Scale bar, 100 µm (b and d). Statistical analysis of protective effects. Living cells were scored. *p < .05. PEDs = proelectrophilic drugs; ARPE-19 = human apical retinal pigmented epithelial cells-19; DMSO = dimethylsulfoxide; HP = hydrogen peroxide; TM = tunicamycin.

An important biological attribute of PEDs is that they offer protection from oxidative stress (Satoh and Lipton, 2007; Satoh et al., 2013). Thus, we examined whether D1 could protect neuronal cells against oxidative stress (Figure 5). As an *in vitro* model of cell death related to AMD (Wada et al., 2001; Kim et al., 2003; Mandel et al., 2009), we exposed ARPE-19 cells to HP (1 mM) for 4 hr in serum-free medium (Figure 5(a) and (b)). In our studies, we found that *ortho-*D3 was about twofold less potent than *para-*D1.

In addition to oxidative stress, we hypothesized that PEDs could also render cells resistant to ER stress by inducing HSPs, as shown previously (Satoh et al., 2011). Hence, we tested if the addition of D1 could protect ARPE cells from ER stress elicited by 3 µM TM. As shown in Figure 5(c) and (d), exposure to TM (3 µM) induced cell death, but D1 (2-5 µM) provided significant protection. D3 was also effective against both oxidative and ER stress, but proved to be a less potent proelectrophilic analog. In addition, although compound D3 afforded protection against TM, it did not activate the HSE. One possibility is that Nrf2 can induce HSPs in addition to phase 2 enzymes without affecting the HSE (Satoh et al., 2011).

### Protective Effects in HT22 Cells

**Figure 6.**
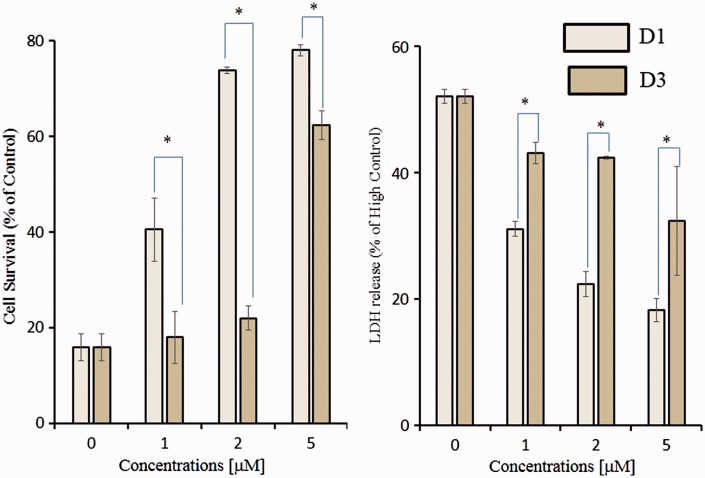
Inhibition of oxidative glutamate toxicity by D1 and D3. HT22 cells were seeded onto 24-well plates at a density of 4 × 10^4^ cells/cm^2^. After a 1-hr incubation, various concentrations of D1 or D3 were added to the cultures. One hour later, 5 mm glutamate was added, and the cells were then incubated for an additional 24 hr. Histogram of MTT assay (a) and LDH release (b) to assess survival and death, respectively, after incubation in various concentrations of D1 or D3 in the face of 5 mM glutamate oxidative insult. Results represent three independent experiments, *p < .05. Results expressed as percentage of maximal LDH released following lysis with 1.0% Triton X-100, designated as *High Control* (b). MTT = 3-(4,5-dimethylthiazol-2-yl)-2,5-diphenyl tetrazolium bromide; LDH = lactate dehydrogenase.

To confirm that *para*-D1 exhibited more potent neuroprotection than *ortho-*D3, we undertook an additional series of experiments examining neuronal cell death in the face of oxidative stress. We examined the protective effects of D1 and D3 against oxidative glutamate toxicity in mouse hippocampal HT22 cells. In these cells, high concentrations of glutamate induce cell death through depletion of glutathione, which is caused by inhibition of the glutamate-cystine antiporter (Satoh et al., 2008a, 2008b). We performed both the MTT assay for cell survival and LDH release assay for cell death. D1 significantly protected cells against oxidative glutamate toxicity, whereas D3 was less potent, as assessed by MTT assay (Figure 6(a)). Again, *ortho-*D3 was about twofold less potent than *para-*D1. These protective effects were confirmed by the LDH release assay (Figure 6(b)).

## Discussion

In the present study, we compared the chemical and biological effects of *ortho*- and *para*-PEDs in providing neuroprotective activity. We performed a series of biochemical experiments, measuring oxidation potential, transcriptional activation, induction of phase 2 enzymes and HSPs, and neuroprotection, to compare the compounds D1 and D3, which share similar chemical structures except that they are *para-* and *ortho-*hydroquinones, respectively. The *para*-electrophilic compound D1 activated neuroprotective signaling pathways to a greater degree than the *ortho*-compound D3. These results are consistent with our CV experiments showing that the sequence of oxidation potential modulation for theses isomers is *para* (hydroquinone) > *ortho* (catechol) > > *meta* (resorcinol) as shown in Table 1. We consistently found that the oxidation potential for the *para*-hydroquinone D1 was lower than that of the *ortho*-hydroquinone D3 (Figure 2). Overall, the *para*-compound exhibited at least a twofold increase in the potency of neuroprotection over the *ortho*-compound. Whether this improvement will be reflected when *para*- versus *ortho*- compounds are compared in *in vivo* disease models remains to be determined in future studies.

In terms of developing clinically tolerated drugs, we sought to learn principles from other recent ventures in successful central nervous system drug development. Along these lines, our development of the FDA-approved drug memantine, an *N*-methyl-d-aspartate receptor antagonist, was in part based on the principle that drugs should interact with their target only during states of pathological hyperactivation and not during normal physiological function (Lipton, 2004, 2006, 2007). Drugs that have been developed using this strategy are designated PAT drugs. PEDs are candidate PAT drugs because conversion to the active quinone form is redox-controlled and thus enhanced by the very oxidative stress that these drugs then counteract (Satoh and Lipton, 2007; Satoh et al., 2008b, 2013). Moreover, in terms of their *druggability*, published work has shown that these PEDs can translocate into the retina and brain of mice and rats at levels sufficient to protect against significant oxidative insults, including light-induced retinal degeneration and middle cerebral artery occlusion (Satoh and Lipton, 2007; Satoh et al., 2008b, 2013).

**Figure 7.**
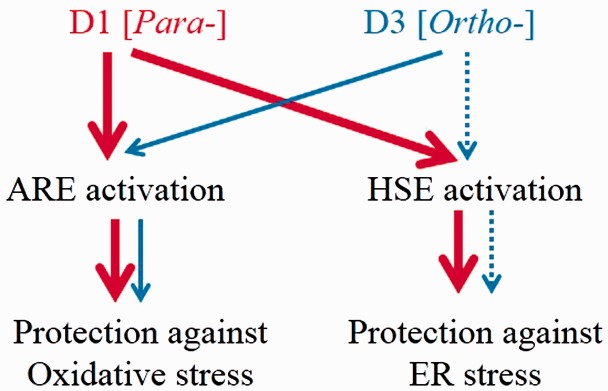
Proposed protective mechanisms of PEDs. Red and blue lines indicate activation of stress response signaling pathways by D1 and D3, respectively. D1 activates both the ARE system and the HSE system, inducing phase 2 enzymes and HSPs, and thus protecting neurons against oxidative stress and ER stress. By also inducing HSPs, D1 reduces protein misfolding, alleviates ER stress, and affords significant resistance to neuronal cells. Compound D3 is a weaker activator of these pathways. PEDs = proelectrophilic drugs; ARE = antioxidant response element; HSE = heat-shock factor response element; HSP = heat-shock protein; ER = endoplasmic reticulum.

Overall, our results support the notion that compound D1 activates both Nrf2 and HSF-1, while compound D3 activates these pathways less potently. Moreover, the measured electrochemical oxidation potentials of these PEDs can be used to predict their activation of protective pathways. The differential ability of PEDs to activate the transcription factors in these pathways is inversely correlated to their potential for oxidation from the hydroquinone to the quinone form (Figure 7). Importantly, the redox state of the cells also affects the ability to transform PEDs to their active quinone form. The Cu^2+^/Cu^+^ redox system regulates oxidative reaction from hydroquinone to quinone (Satoh et al., 2009; Wang et al., 2010), and this system is highly influenced by the availability of electron acceptors in the cell; under pathological conditions, such electron acceptors are represented by reactive oxygen species (Satoh et al., 2009; Wang et al., 2010). Redox state-dependent regulation of quinone formation dictates that PEDs, such as CA and D1, are PATs in the sense that they are converted from the hydroquinone to the quinone by the very reactive oxygen species that they then combat via transcriptional activation (Lipton, 2004, 2006, 2007). Accordingly, activated PEDs *S*-alkylate critical cysteine residues on KEAP1 and HSP90, leading to activation of the transcription factors Nrf2 and HSF-1, respectively (Satoh et al., 2011; Zhang et al., 2011). This approach thus represents a novel strategy against neurodegenerative disorders, providing disease-modifying electrophilic drugs in the context of pathological insult.

For many years, the herb rosemary has been reported to manifest antioxidant and anti-inflammatory activity. We have previously shown that CA, present in rosemary extract, crosses the blood–brain barrier to exert neuroprotective effects by upregulating endogenous antioxidant enzymes via the Nrf2 transcriptional pathway (Rezaie et al., 2012; Satoh et al., 2013). The antioxidant and neuroprotective activities in retinal cell lines exposed to oxidative stress and in a rat *in vivo* model of light-induced retinal degeneration suggest that PEDs, such as D1 and CA, may potentially have clinical application to retinal diseases, including AMD and retinitis pigmentosa, in which oxidative stress is thought to contribute to disease progression (Rezaie et al., 2012).

In conclusion, our findings suggest that *para*-hydroquinones are both more potent and efficacious than their *ortho*-hydroquinone homologues in terms of transcriptional activation, induction of phase 2 enzymes and HSPs, and neuroprotective effects against oxidative and ER stress. Nonetheless, other factors that determine druggability, such as pharmacokinetics, bioavailability, stability, metabolism, and translocation into the brain, will also be important in determining which structures are most effective *in vivo*. Critically, however, this is the first report to our knowledge to demonstrate that positional isomers of proelectrophilic hydroquinones are an important determinant in the activation of Nrf2- and HSF-1-mediated stress responses, and the disparate effect of these isomers is closely tied to their oxidation potentials.
